# Properties and Behavior of Sandy Soils by a New Interpretation of MICP

**DOI:** 10.3390/ma18040809

**Published:** 2025-02-12

**Authors:** Masaharu Fukue, Zbigniew Lechowicz, Catherine N. Mulligan, Seiichi Takeuchi, Yuichi Fujimori, Kentaro Emori

**Affiliations:** 1 Japanese Geotechnical Association for Housing Disaster Prevention, 1622, Oshikiri, Shimizu-ku, Shizuoka 424-0008, Japan; fukue@scc.u-tokai.ac.jp; 2 Department of Geotechnical Engineering, Institute of Civil Engineering, Warsaw University of Life Sciences, Nowoursynowska 159, 02-776 Warsaw, Poland; zbigniew_lechowicz@sggw.edu.pl; 3 Department of Building, Civil and Environment Engineering, Concordia University, 1455 de Maisonneuve Blvd. W., Montreal, QC H3G 1M8, Canada; 4 Fudo Tetra Co., 7-2, Koami-Cho, Nihonbashi, Chuo-ku, Tokyo 103-0016, Japan; seiichi.takeuchi@fudotetra.co.jp; 5 Chubu Sokuchi Research Institute Co., 801-1 Konami, Suwa City 392-0131, Japan; fujimori-yuichi@chubusokuchi-lab.co.jp; 6 Sanko Kaihatsu Co., Ltd., 1320 Gokanjima, Fuji City 416-0946, Japan; k.emori@sankoukaihathu.co.jp

**Keywords:** MICP process, optical density (OD), cell viability, carbonate formation rate (CPR), OD−CPR relationship, sandy soils

## Abstract

Research on MICP technology for ground improvement began in the early 2000s, and since then, it has been considered as innovative research. The field of applications is showing signs of expanding from sandy soil stabilization to remediation. However, the research has not always progressed, because it is extremely difficult to evaluate the ability (viability rate) related to microorganisms and how to handle them quantitatively. In fact, this problem hinders the consensus of research results in terms of quantitative evaluation of microorganisms and the cross-comparison (evaluation) and use of MICP technology research. The crucial disadvantage of using bacteria is that their properties are not constant due to changes over time and in the surrounding environment. Therefore, for engineering purposes, we used the carbonate formation rate (CPR), instead of urease activity, as a function of the microbial mass (OD) with viable bacteria. Thus, the standard OD−CPR relationship was defined experimentally, and the estimation method of viability was established. The required amount of microorganisms for testing was given by OD*, and the relationship “OD = Rcv OD*” was defined to convert from OD* to OD. Rcv was defined as the viable bacterial rate. It was found that the Ca^2+^/OD ratio controls the inhibition behavior in MICP. At a Ca^2+^/OD ratio of >8.46 M, then inhibition occurs, while at Ca^2+^/OD = 8.46 M, CPR = 8.46 OD and the CPR is proportional to the viable OD, Rcv, and OD*. We show that it is possible to perform an experiment using OD* with aged bacteria, obtain Rcv from the standard OD−CPR and OD*−CPR relationships, convert OD* to OD and to perform a unified evaluation without actually determining the viability rate.

## 1. Introduction

Microbial-induced carbonate precipitation (MICP) has been studied as one of the next-generation technological developments, because it is an eco-friendly natural technology learned from nature. The technology uses biominerals as binding materials, which has been introduced in the fields, such as sand stabilization [1], loess surface erosion control [2], reduction of liquefaction potential [3], scour protection around monopile foundation [4], increase in pull-out response of concrete pile [5], erosion protection of clayey soils [6], wind erosion control of desert soil [7], development of biogrout injection techniques [8], liquefaction resistance [9], stabilization of foreshore slopes [10], and ground improvement by biogrout [11]. In remediation technology, the application of MICP is also becoming increasingly popular in the fields of soil and water purification [12], solidification and remediation of lead−zinc tailings [13], strontium incorporation [14], remediation of uranium (VI) [15], remediation of heavy metals [16,17,18,19,20], biomineralization [21,22,23], remediation in mines [24,25,26,27], etc. In these areas, there are a number of researchers and many journal submissions published and in progress.

However, these achievements are still insufficient because these developments are relatively new since 2000. In addition, since MICP research is highly interdisciplinary, the collaboration of many researchers with different expertise may be slowing progress.

At present, there are essentially three problems for applying MICP in geoenvironmental engineering. Bacteria are living organisms, but they can be damaged over time due to age, temperature, pH, etc. Thus, their properties and behavior in the MICP process can change [28]. This means that the experimental results obtained depend on the viability of cells. This results in the decrease in optical density of cells which will cause inhibition or retardation in MICP [29].

Until now, the evaluation of microorganisms in MICP has been performed by determining the optical density (OD) of microorganisms in the culture medium. For example, the urease activity also has been evaluated by obtaining the optical density of bacteria, OD_600nm_, which has been assumed as the density of the viable bacteria solution [29,30,31]. However, the optical density determination cannot distinguish between live and dead bacteria. In addition, the density of viable bacteria changes with aging, cold and heat shock, and various other elements. Thus, it is noted that caution should be exercised in the use of optical density.

In reality, despite these shortcomings, the use of OD is common. A possible shortcoming is that the inhibition and retardation effects of Ca^2+^ and OD on MICP are not well understood [29]. According to Fukue et al. [28], when the Ca^2+^/OD value is greater than a certain value, then inhibition due to Ca^2+^ occurs. Therefore, when the OD value decreases with aging or a change in a factor, inhibition will occur under the same concentration of Ca^2+^. The critical Ca^2+^/OD value can be confirmed using only OD which is viable optical density, as was achieved in Fukue et al. [28]. If the descriptions above are correct, the comparison and identification of the data made in the past based on the conventional OD values are uncertain.

To use unified OD values in engineering purposes, Fukue et al. [28] found a method to convert from the apparent OD* to viable OD, in which the viable OD is defined as OD = Rcv OD*. The viable bacteria rate (Rcv) plays a role in cell viability. In addition, both OD and Rcv OD* are related to the carbonate precipitation rate (CPR) which is defined as the experimentally obtained standard OD−CPR relationship. The standard OD−CPR relationship is also used to judge if inhibition or retardation occurs. The OD*−CPR relationships have been obtained to investigate the capacity of bacteria for carbonate precipitation [29].

At present, the concentration of bacteria is not considered in the chemical reaction of the MICP process. In other words, concepts of mixture design as used in construction materials have not been applied. To solve this problem, the blending design method was established [28,29], using a unique (standard) carbonate precipitation (CPR)−OD relationship. At the same time, the conversion was enabled from an experimentally tentative optical density (OD*) to OD. Using the OD conversion and blending design of the biocement solution (BCS), the inhibition and retardation behavior in MICP were interpreted well, because experimental results obtained using different OD* were able to be combined and compared to the OD conversion [29]. It is highlighted that the optimum blending design of the BCS becomes possible. In other words, CPR has been able to be controlled with bacterial concentration in the BCS. By making full use of these methods, it is likely that significant advances and developments in MICP technology will be obtained. In this study, the objective is to show a new approach and to interpret the mechanical properties and behavior of soils in MICP processes, which have been unknown. While this study seems to be only from a macroscopic point of view, it is based on the microscopic point of view, both theoretically and experimentally for more than 15 years.

## 2. Materials and Methods

In this study, two typical granular materials, natural limestone [30] and MICP-treated fine sand [11], were examined in terms of dry density (ρ_d_) and unconfined compressive strength (UCS), and the strength properties on the elemental tests and the behavior of the biomineralization processes were compared.

The ρ_d_−UCS fitting curves for Ryukyu limestone were obtained by Kogure et al. [30]. The Ryukyu limestone consists of solidified sediments of coral sand and gravels, with some binding between particles. The formation has not been well understood. However, the lysis-reprecipitation of coral sand and gravel particles and/or foraminifer and coccolith have possibly solidified from the granular to solid materials, similar to general marine sediments [31,32,33]. The increase in dry density of the limestone may be due to the binding materials (biominerals).

As a result, the UCS of limestone was expressed with dry density, ρ_d_, as:

UCS = 2.2 × 10^−2^ ρ_d_^8.8^      (MPa) (1)

The square sectional area (*d* × *d*) and 2*d*-height specimens were used for the UCS measurement, and the effects of *d* were investigated using various *d* values. The *d* changed from 10 to 100 mm with 5 stages, i.e., 10, 25, 50, 75, and 100 mm. The numbers of specimens increased with decreasing *d*, corresponding to 2, 5, 8, 10, and 20, respectively. As a result, the coefficient of determination R^2^ obtained was 0.78 [30]. However, in Equation (1), the role of carbonate content is uncertain.

On the other hand, artificial MICP was applied to a large-sized fine sand embankment by van Paassen [11]. The ρ_d_−UCS fitting curve was obtained by:

UCS = 4 × 10^−5^ exp(6 ρ_d_)      (MPa) (2)

where the coefficient of determination R^2^ is 0.84. In this test, the biogrout solution was infiltrated laterally, and it was found that carbonate content apparently increased the UCS of fine sand and that the dry density increased with increasing carbonate contents. The increased dry density was assumed to be equal to the increased biogrout (biocement) in this analysis. It is important to note that the UCS can only be developed by bonding particles for granular materials. There is no doubt that the binding materials were precipitated carbonates. It was noted that the R^2^ of the C−UCS relationship obtained was lower than that in Equation (2) [11].

Next, the similarity in solidification properties and behavior of the two types of granular materials under different solidification mechanisms was examined to understand essential binding mechanisms due to carbonation for granular materials.

**Figure 1:**
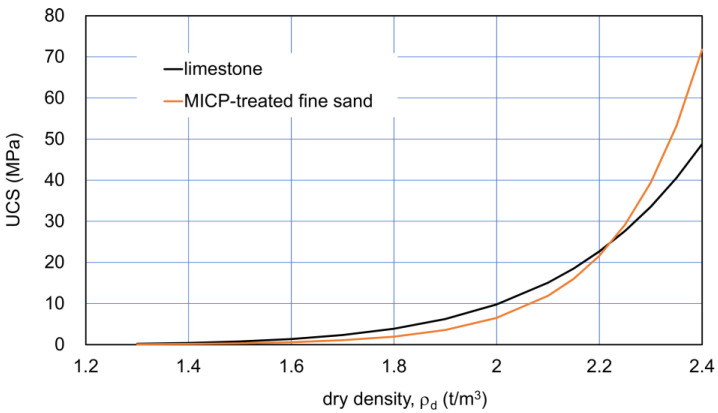
Dry density−UCS fitting curves for Ryukyu limestone [30] and for MICP-treated fine sand [11].

The comparison between the ρ_d_−UCS relationships for the limestone and fine sand is presented in Figure 1. It is noted that the curves lower than 1.7 t/m^3^ were not based on actual data. Nevertheless, the extension curves by Equations (1) and (2) were used to discuss both materials with a relatively low UCS. It is because these low ranges in the UCS are very important for material improvement.

In Figure 1, the effects of *C* on UCS were hidden, because no C axis is presented. The R^2^ values were 0.78 and 0.84 for the limestone and fine sand, respectively. The initial states for the two curves were assumed as presented in Table 1. The assumed values as the typical examples of sediments were not extreme for the comparison. In general, the factors affecting the minimum and maximum densities are the geometrical factors of particles, such as grain size distribution and grain shape, as well as the density of particles. The maximum void ratio of calcareous sediments is usually higher than that of ordinary sand (Table 1), in which it is not necessary to consider the density of particles.

Because of the differences in the textures of particles, formation mechanisms to rocks, physical and chemical properties, and behavior between natural limestone and MICP sandstone, it seems that the comparison may be meaningless. However, it is possible that both materials have become solidified as a result of carbonate precipitation, regardless of the mechanism [32,34]. Further comparison showing a similarity of the two curves in Figure 1 was examined in terms of the newly defined relative density.

In geotechnical engineering, the unification in properties among different types of granular soils are often examined using relative density. In this study, the definition of “modified relative density Dr*” was changed from the conventional definition of granular soils, because the transformation from granular state to rock (solid) materials was regarded as continuous. Therefore, the minimum density e_min_ was assumed as zero.

Accordingly, the modified relative density of materials consisting of granular to rock states, Dr*, was defined by:

(3) $${Dr}^{*}=\frac{e_{max}-e}{e_{max}}$$

**Figure 2:**
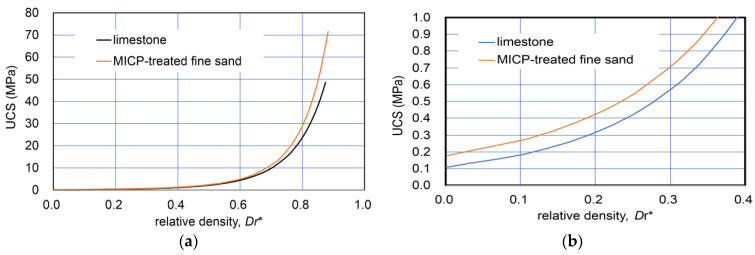
Comparison of Dr*−UCS relationships for limestone and MICP-treated fine sand: (**a**) wide range of Dr* (0–0.85); and (**b**) a narrow range of Dr* (0–0.4).

The comparison with modified relative density−UCS relationships converted from those in Figure 1 is shown in Figure 2. Figure 2b indicates that the two curves for a wide range of dry density were close together and that limestone showed a higher UCS than that of MICP-treated fine sand. The details are discussed later in further data for sandy soils.

Figure 1 and Figure 2 indicate that the natural diagenesis of limestone and MICP processes on fine sand may show similar properties and behavior despite their different natures. In other words, their varied nature and solidification process can be primarily described in terms of strength hardening due to both the increases in carbonate and dry density. This will be discussed in the experimental results for sandy soils later.

### 2.1. Sandy Soils Used in MICP

To investigate the C−UCS relationship in MICP for various granular soils, six different types of sandy soils (obtained from Tokai Sand Co., Omaezaki City, Japan. http://tokaisand.co.jp/archives/145 accessed on 5 January 2025) were used, which are as follows:

- Vietnam sand, crushed sand with high-electrostatic charges;
- Coarse sand with a low uniformity coefficient;
- Fine sand a with high liquefaction potential;
- Medium sand;
- Fine sand b with high liquefaction potential;
- Fine sand c (densified fine sand b);
- River sand with a high uniformity coefficient.

The numerical data are presented later using the six types of sandy soils (seven different specimens).

### 2.2. Problems in UCS Measurement for Loose Sandy Soils

The block samples of MICP-treated sandy soils were used for the mechanical tests in this study. The test method will be described in Section 2.3.1. Two types of specimens were used, depending on the test types. Unconfined (uniaxial) compression (using equipment from KS-12210, Tesco Ltd., Arakawa-ku, Japan), and triaxial compression tests (with equipment from KS-12230, Tesco Ltd., Arakawa-ku, Japan) were performed on cylindrical specimens trimmed from the block samples by Japanese standard test methods (JIS A 1216 and JGS 0521, respectively). The preparation method of the specimens was the same as used in geotechnical engineering, except for the handling difficulty. On the other hand, a pocket penetrometer test was performed directly on the block samples.

Since the binding carbonates built crystalline skeleton structures, the failure strains in limestone and MICP under uniaxial compression was very small, which was described as the failure of brittle materials.

**Figure 3:**
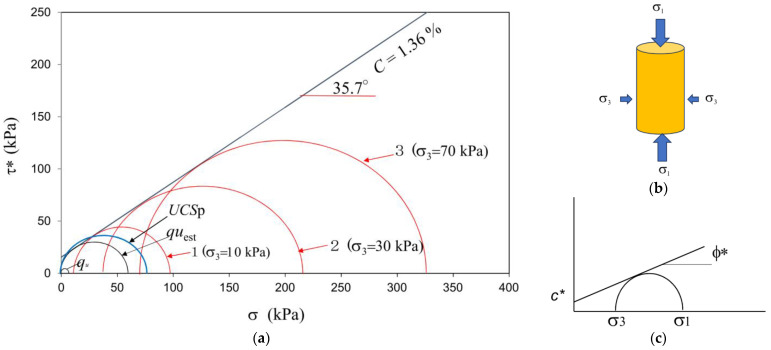
(**a**) Various Mohr‘s circles in triaxial compression; (**b**) specimen in triaxial compression; (**c**) the Mohr−Coulomb failure criterion. * Denotes the values for MICP treated soils.

If the specimens of very loose sandy soils were prepared in MICP, the UCS would be extremely low in comparison to the unconfined compressive strength estimated from the triaxial compression test, as shown in Figure 3. The unconfined compressive strength was expressed by qu, while UCSp was measured by the pocket penetrometer obtained from CL-700, SOIL TEST INC. CHICAGO USA and used according to ASTM WK27337. The difference between the two values was apparent. Note that the Mohr’ s circle under a low σ_3_ stress seemed to be the appropriate size.

On the other hand, UCSp, qu_est_, and Mohr’s circles 1, 2, and 3 showed reasonable Mohr’s circles with the Mohr−Coulomb failure criterion, as shown in Figure 3. Thus, only qu was extremely low in comparison to the other experimental data under the triaxial failure criterion.

**Figure 4:**
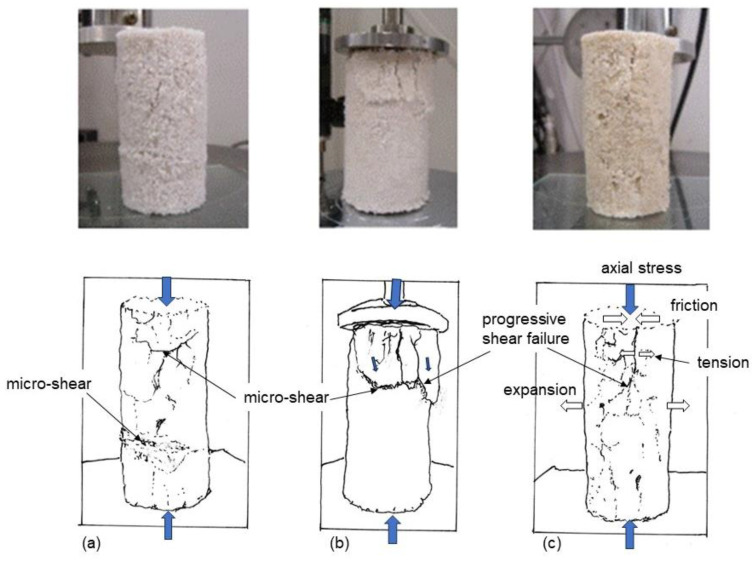
Micro-failure patterns in tension for loose MICP-treated sandy soils in uniaxial compression tests. (**a**–**c**) indicate three MICP-treated specimens.

It was considered that the specimens in the uniaxial compression tests failed in tensile forces, micro-failures, as shown in Figure 4. The failure patterns were dissimilar from general shear failure in the ordinal soil specimen in the triaxial compression test. This resulted from the brittle properties of MICP-treated loose sands.

The failure types depend on the porosity of soil specimen which may concern the inhomogeneity of structure consisting of particle arrangement. The looser the sand, the more complicated the properties and behavior became, as shown in Figure 4. The failure patterns could be expressed in terms of micro-shear or progressive tensile failures, as shown in Figure 4a,b, or tensile failure, which was more explainable theoretically, as shown in Figure 4c. In addition, it is possible that UCS (solidification) may affect the failure pattern. At present, it is difficult to predict failure patterns of MICP-treated loose sand in uniaxial compression as it is different from the ordinary sand and rock.

The results shown in Figure 4 indicate an example of brittle failures of MICP-treated soils of low density that can be avoided by applying a low lateral confinement stress.

**Figure 5:**
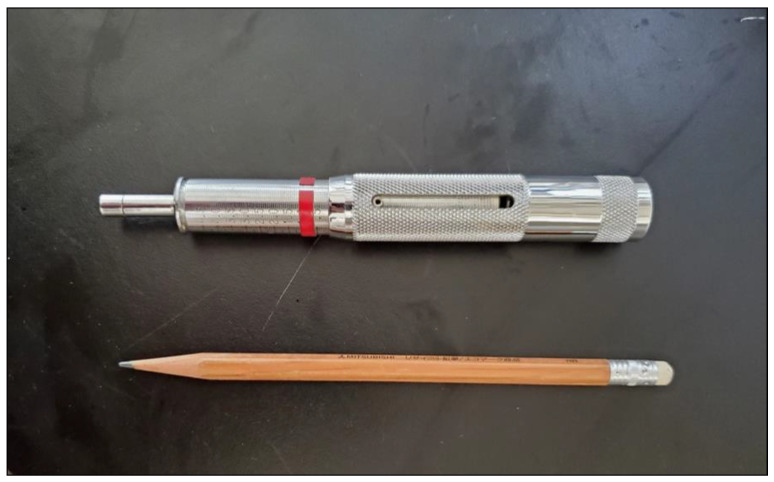
Pocket penetrometer (ASTM WK27337).

The pocket penetrometer used is shown in Figure 5. The test method is simple. Some discussion on the test method and applications have been published [35,36,37]. The disadvantage in the use of the penetrometer is a limited maximum UCS, i.e., 460 kPa, but it is sufficient for the MICP study on low-density sandy soils with liquefaction potential.

In summary, the pocket penetrometer is useful to measure UCS for low-density MICP-treated sandy soils from the viewpoints of time and cost saving. The results obtained may be a little influenced by confined lateral pressure during penetration of the tip into soil. However, it seems that the developed confined pressure, due to the penetrating effect by the penetrometer’s tip, was neglected or at most less than 10 kPa. It is noted that liquefaction potential is of the most concern for this type of sand.

### 2.3. Experiments

#### 2.3.1. Preparation of Specimens of Seven Types of Sandy Soils

To investigate the carbonate (C)−UCS relationships, the six types of sandy soils were loosely packed in the three boxes made of styrofoam. To examine the effects of densification, fine sand B was also densified into three styrofoam boxes. In the BCS, 0.3 M Ca^2+^ (obtained from Tokuyama Corporation,1-1, Shunan City, Japan), 0.3 M urea (supplied by Kanto Chemical Corporation Inc., Chuo-ku, Japan), and NO-A10 strains (obtained from Life Engineering Co., Shimizu-ku, Japan. https://life-engineering.jimdoweb.com accessed on 5 January 2025) were contained. The OD value of NO-A10 strains was unknown, and the amounts of bacteria were based on the experiment. The initial density of soils was calculated from mass, volume, and water content of air-dried soils. The surfaces of the packed soils were covered by unlaid cloth and netting. The drainage was allowed from the bottom side with a tube. The injection volume of the BCS was approximately 1.2 -fold that of the pore volume. The injection of the BCS was conducted by spraying without considering the homogeneity of the ultimate C value, because the study required the deviation of C values through the specimens. Since the thickness of specimens was relatively thin, the injection was conducted after the production of white suspensions and crystalline carbonate began in the BCS.

**Figure 6:**
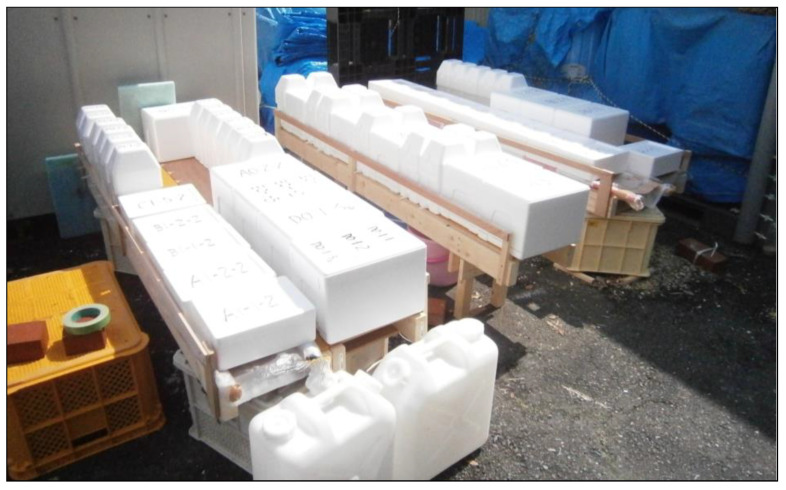
Curing setup of MICP experiments.

The three boxes for one soil sample were used to vary the number of injections. The number of injection cycles was varied from 1 to 3. After the injections, the specimens were rinsed with sufficient tap water and left for at least for one month. The curing setup is shown in Figure 6.

#### 2.3.2. Carbonate Content Determination

The carbonate contents (with equipment from Life Engineering Co., Shizuoka, Japan. https://life-engineering.jimdoweb.com accessed on 5 January 2025) were determined by measuring the CO_2_ gaseous pressure induced in a pressure cell obtained from DMC-203N11, No.92AF 015, Okano Works Ltd., Neyagawa City, Japan. https://okanoworks2.com/products/pressure accessed on 5 January 2025), with 3 N HCl (obtained from Wako Pure Chemical Industries, Ltd., Chuo-ku, Japan). The CaCO_3_ contained in the soil samples was converted to the mass of CaCO_3_ using a linear CaCO_3_ gaseous pressure calibration curve [38]. The method is simple and easy to measure using several grams of the dry MICP-treated sample. Generally, it only takes a few minutes of operation per single sample, except for the preparation of samples, i.e., drying of samples in the oven and weighing.

The carbonate content measured on MICP-treated soil was expressed by:

C_t_ = CaCO_3_/dry soil sample × 100       (%) (4)

where C_t_ is the total of natural carbonate after MICP treatment.

In fact, a natural soil sample usually contains a small amount of CaCO_3_ as soil particles [31,33], which does not contribute to the strength. Therefore, to eliminate the natural CaCO_3_, a similar test should be performed using the original natural soil sample. To present the carbonate content alone by MICP, the naturally contained CaCO_3_ content must be deducted from C_t_ in Equation (4).

## 3. Test Results

### 3.1. Carbonate Content and UCS

**Figure 7:**
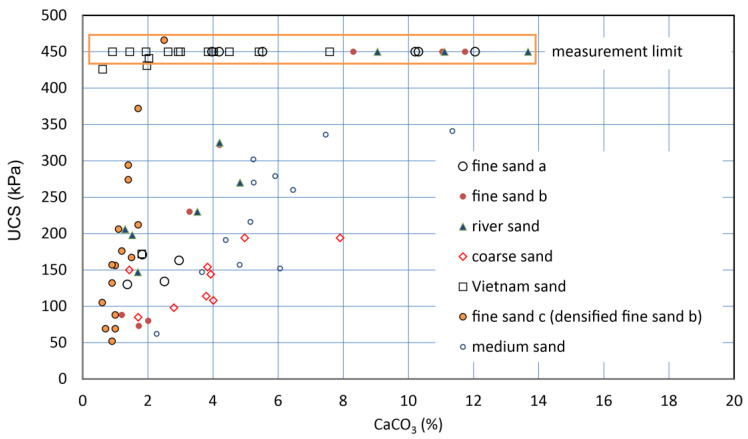
C−UCS relationships for various sandy soils.

The UCSs of six soil samples, i.e., seven types of specimens, were measured with the pocket penetrometer. The UCS increased with carbonate content. As a result, for the respective specimens, the higher the carbonate content, the greater the UCS. The results showed that most of the UCS values increased linearly with increasing carbonate content (Figure 7). This type of C−UCS relationship has been presented and discussed by many studies. However, there is no consensus based on only the C−UCS relationships, as they are dependent on the soil type.

The soil properties and a-values obtained are presented in Table 2. Note that the a-values were estimated as the slopes of the fitting *C*−UCS curves by:

UCS = a C      (kPa)(5)

However, the a-values might be underestimated, because some of the measurements were possibly influenced by the disturbance of samples. For example, for Vietnam sand, a-values seemed to between about 180 to 690 kPa/%. This may result from the very high electrostatic charges of the Vietnam sand particles (see Figure 7).

Therefore, the a-value that can be specific for a soil type is significant. In this study, the a-value was defined to be a distinct mechanical behavior in MICP. In addition, the dependency of various dry densities on a-value will be discussed later.

The maximum and minimum dry densities of soil samples were measured by the method in Japanese Industrial Standard, JIS A 1224. Theoretically, the initial dry density must accord with the minimum dry density. However, the initial dry density of soil specimens was sometimes lower than that of the minimum dry density because the specimens contained macropores (Table 2). D_max_, D_60_, D_30_, and D_10_ represent the maximum particle diameter and the particle size at which 60%, 30%, and 10% of the particles are smaller, respectively.

### 3.2. UCS as a Function of Carbonate Content and Dry Density

One of the features of MICP is that there is no change in soil volume. This is due to the low-pressure infiltration of BCS. On the other hand, MICP must increase the dry density due to the precipitation of solid carbonates.

In marine sediments, the dead bodies of foraminifera and coccolith are always present, which contribute to the binding of the sediment particles. This is also applicable to natural limestone formation from coral sand and gravel [35,36]. In any case, the build-up of a skeleton structure in granular soils including coral sediments requires particle-to-particle bonding, which is due to the MICP process and dissolution and reprecipitation under the change in micro-circumstances, such as partial pressure of CO_2_, redox potential, pH, and Ostwald’ ripening and/or sintering under appropriate conditions [33,39,40,41,42,43,44,45].

Assuming that the carbonates induced by MICP and metamorphisms increased the dry density of sediments, carbonate content C and dry density ρ_d_ relationship was given by:

(6) $$\rho_{d}*=\rho_{d}\;(1+\frac{C}{100})\;(t/m^{3})$$

where ρ_d_* is the dry density after MICP or carbonate diagenesis, ρ_d_ is the initial dry density, and C is the carbonate content increased by MICP or carbonate diagenesis.

**Figure 8:**
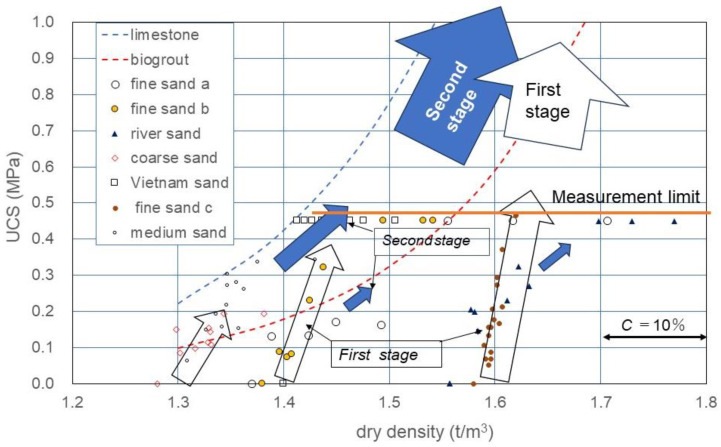
UCS as a function of C and ρ_d_, assuming that dry density was increased with increased carbonate precipitation.

Figure 8 shows the ρ_d_*−UCS relationship obtained using Equation (6). In Figure 8 as described earlier, the linear C−UCS relationship obtained was clearly positioned in the ρ_d_*−UCS relationship, as well as in Equation (6).

Though the UCS measured was plotted on the ρ_d_ axis, the carbonate content was converted into the incremental dry density.

### 3.3. Definition of the First and Second Stages in the Carbonation Process

Table 2 and Figure 8 show that the UCS in MICP-treated soils varied with soil type including grain size, dry density, etc. Furthermore, it was shown that for the incremental rate of UCS with carbonate content C, the a-value depended on the initial dry density (Figure 8). This fact can easily be explained with the correlation between the number of contacts between particles and dry density, because the a-value increased proportionally with the increased contact points between particles due to the increase in dry density.

The initial effects in the MICP process were described as the rapid bonding at the contact between particles because the binding started with zero strength. The denser the skeleton structure, the higher the UCS. It was experimentally found that the rapid increase in UCS was proportional to the C value. In this study, this initial process was defined as the first stage in MICP, as expressed by the empty arrows in Figure 8.

After the skeleton structure was developed to some extent, newly induced carbonates covered the surface of soil particles. However, they lacked the strengthening power of the skeletal structure. The slow MICP process after the first stage was defined as the second stage in MICP, as presented by the filled arrows in Figure 8. It is noted that there might be an infinite number of processes with different soil types in MICP. For example, with respect to the initial dry density of a soil, an infinite MICP process was considered.

### 3.4. Initial Dry Density and a-Value

It was indicated that the a-value can be one of the mechanical factors in various MICP-treated soils. From Equation (4), the a-value was given by:

a = UCS/C       (kPa/%)(7)

**Figure 9:**
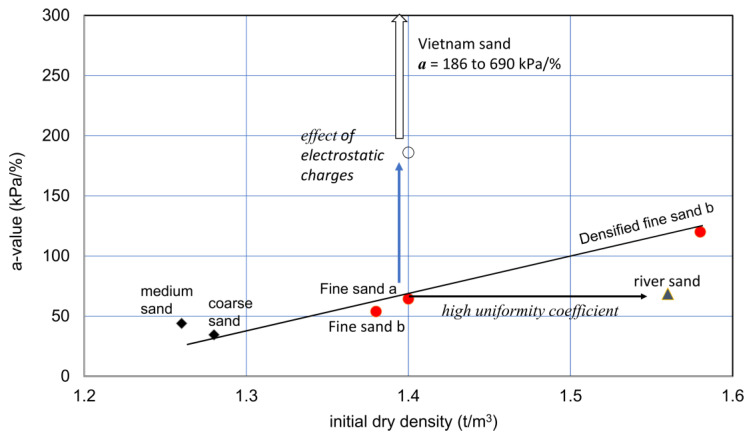
Initial dry density and a-value relationships for various sandy soils. The vertical arrow indicates the increase in a value due to the special action due to particle’s surface forces, under a con-stant distribution of particle sizes. On the other hand, the horizontal arrow shows a constant a-value for particles from ordinary or averaged particle distribution at a given dry density.

The a-values of the seven types in the test series are presented in Table 2. The initial dry density and a-value for the various sandy soils are presented in Figure 9. It seems that the plots deviate widely. This indicates that the soil samples used had properties previously mentioned. In other words, Figure 9 indicates the different properties expressed by the a-value at the zero potential energy state (like at the initial dry density). The initial void ratio or initial dry density of soils affected the e−log p relationships for the soil, which was explained by the concept of the equation of state in soils [43].

The extremely high a-value in Vietnam sand, i.e., 186 to 690 kPa/%, may result from the effects of high electrostatic charges of particles. The relatively low a-value for river sand may be attributed to its high uniformity coefficient (Table 2), in which relatively greater particles caused a relatively high density without increases in the a-value.

The effect of compaction was examined by comparing the original (fine sand b) and compacted specimens (fine sand c). The result suggests that there may be a unique relationship between dry densities and a-values for similar granular soils with a similar range in uniformity coefficients. Therefore, unless soils are special like Vietnam sand and river sand, the initial dry density−a-value relationship for ordinary granular soils may not differ widely. This needs to be investigated further.

## 4. Discussion

### 4.1. The First and Second Stages in the MICP Process

**Figure 10:**
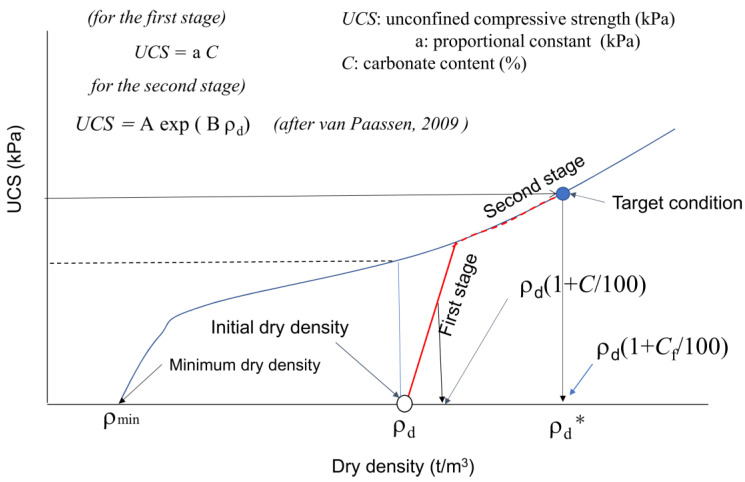
Definition of the first and second stages in the MICP process [11].

In engineering purposes, if the initial dry density is known, the design (target) strength UCS can be expressed by a target C-value in Figure 10. If the target C or UCS is in the first stage, both values are expressed by UCS = a C. On the other hand, if the target C or UCS in the second stage, the second stage must be evaluated or estimated. In the case, a continuous increase in the C value results in a non-linear C−UCS relationship, as illustrated in Figure 10. The non-linear behavior results from the transformation from the first to second stages in MICP. Note that there must be an inflection in the slope for the ρ_d_−UCS curve, as illustrated in Figure 10.

It is unknown if the two second stages started from different first stages for similar soil types. The skeleton structures of the two first and second states are apparently different because the respective skeletons are built at the initial dry density. That is why the a-values for the two different first stages are different. In other words, the second stages will keep their respective skeleton structures from the first stage, and the pore spaces will continuously be filled with carbonates by precipitation.

In Figure 10, the second stage is illustrated based on Equation (2) [11], which can be recognized as an example for the general expression of the second stage of MICP in fine sand. In summary, when the effects of carbonate content on UCS or other strength factors are investigated, the dry density should be incorporated. In the design of MICP, C and ρ_d_ should be considered as factors of strength. Therefore, to avoid confusion, it is important to distinguish the first and second stages in MICP. Note that in this study, the a-value was only defined in the first stage. At present, the estimation (fitting curve) by van Paassen [11] will provide an index for the second stage of fine sand.

### 4.2. Conversion Between C and the CPR

The CPR and C terms have different definitions. The CPR is precipitated carbonate (M) in the BCS, while the C value (%) is carbonate content defined as the mass ratio of CaCO_3_ by soil particles. Both values are conveniently used for individual purposes. Thus, the CPR is used in the case of the BCS without the involved soil, and the C value is always used in the case of UCS when soil is involved.

In procedures of engineering design, carbonate content C (%) is given based on the design strength, e.g., UCS. If the a-value of the soil is known, the C value is predicted by UCS/a. Then, the C is theoretically converted to the carbonate precipitation rate, CPR, in the BCS [28].

When bacteria are mixed with the BCS components such as urea, the hydrolysis of urea is initiated. The hydrolysis occurs in the cell and produces 2NH_3_ and CO_2_ extracellularly. Ca^2+^ reacts with CO_3_^2−^ from the CO_2_ produced by the hydrolysis. In the optimizing case of the Ca^2+^/NO-A10 ratio, the entire process from the mixing of bacteria with other components to the complete precipitation of carbonates requires from approximately at least 30 min to 48 h [28,29]. Therefore, various physical and chemical phenomena occur, such as dispersion of the components, development of a Ca^2+^ diffuse double layer, aggregation of bacteria with the Ca^2+^ diffuse double layer [44,45] and crystal growth, adsorption of the aggregates on soil particles, and settlement of the aggregates.

**Figure 11:**
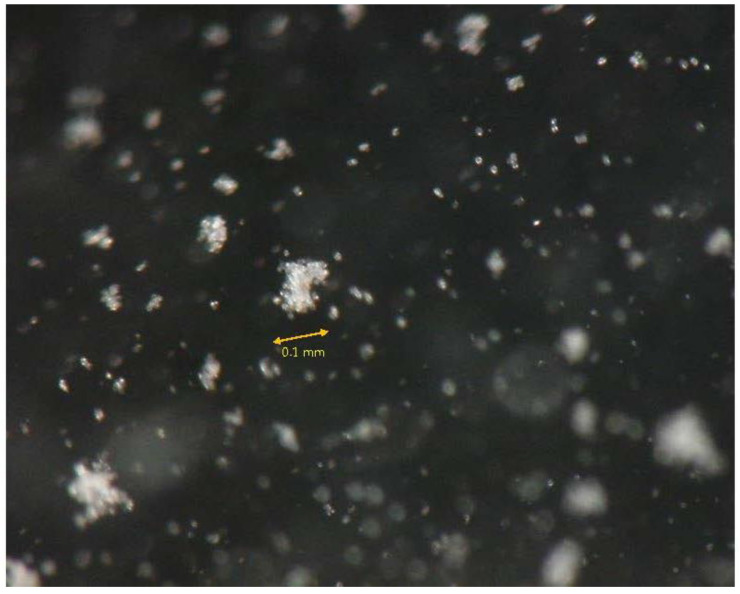
Digital image of aggregation, adsorption, and crystalline in the MICP process.

Figure 11 shows a segment of a video depicting the BCS in a glass tube, including the movement of bacterial aggregates with the Ca^2+^ diffuse double layer by convection. Aggregates adsorbed on the wall of the glass tube, and crystalline growth formed in aggregates. In this case, the video was shot using a digital microscope for approximately 40 min after the BCS was prepared. Accordingly, the bacteria with Ca^2+^ diffuse double layers and the aggregate shapes could not be recognized with the naked eye. The growth of carbonates started around the cells which were seen as cloudy suspensions. It took generally more than 30 min from the mixing of the BCS.

Thus, the organic reaction is not as rapid as the inorganic reaction. For engineering purposes to produce good-quality cementation effects, a slow reaction is required which may contribute to adsorption on aggregate-soil particles.

Bacteria with negative surface charges can be adsorbed on positively charged glass, while negatively charged soil surfaces can adsorb the bacteria with Ca^2+^. Therefore, the beginning adsorption behavior of bacteria with or without Ca^2+^ may be different in a glass tube or soils. However, for MICP, Ca^2+^ is a good binder between soil particles and bacteria.

**Figure 12:**
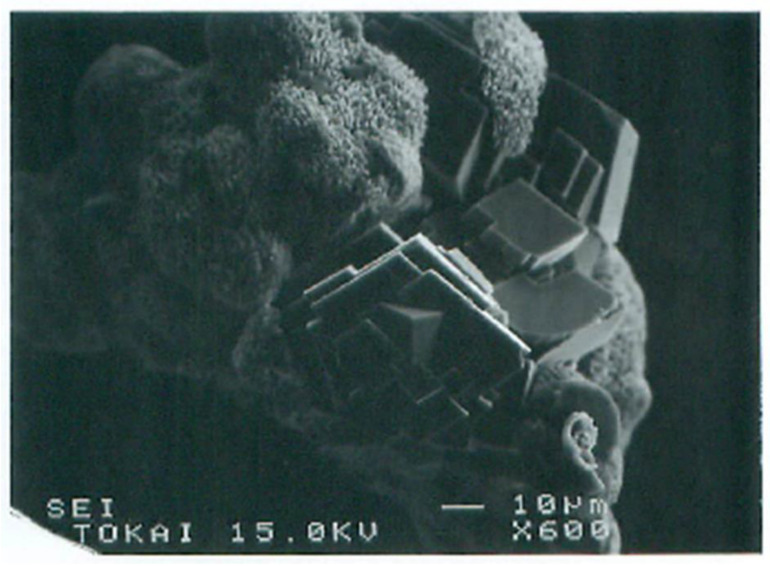
SEM images of calcite growth from amorphous aggregates formed in the original bacteria−Ca^2+^ system in the BCS.

**Figure 13:**
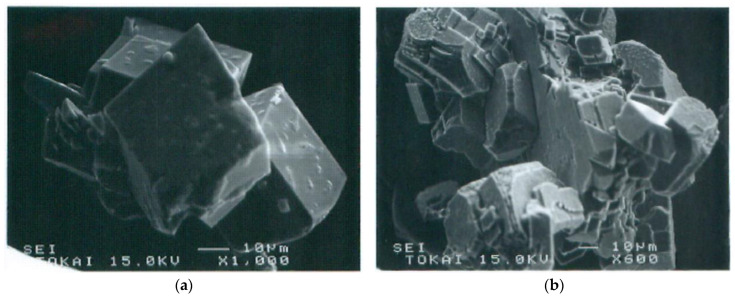
SEM images of ultimate single and polycrystals in MICP: (**a**) single crystals of calcite; (**b**) polycrystals of calcite.

Adsorbed aggregates could grow by absorbing advected aggregates. Crystalline growth depended on the size of aggregates. The precursor of crystals was usually amorphous which then produced crystalline forms in the aggregates (Figure 12). Ultimate crystals were various types of calcites. If only Ca^2+^ was used as a metal element, single crystals were sometimes produced, but they were usually polycrystals (Figure 13).

According to the application of the adsorption theory [46,47], bacterial adsorption seems to be simply expressed by the Langmuir and Freundlich or modified theories. However, in MICP, the adsorption of bacteria on soil particles occurs as bacteria aggregates with Ca^2+^ and partial amorphous and crystalline CaCO_3_. Under the ideal blending of the BCS [28], the formation of the complex aggregates may depend on at least the concentrations of bacteria and initial Ca^2+^, flow velocity of the BCS, and soil dry density.

In this study, a concept based on the bacteria-dependent CPR was used [28] and was presented by Equation (8).

CPR = 8.46 OD − 17.633 (OD)^2^       (M) (8)

If after an elapsed time the urease activity started is sufficient to produce the full amount of carbonate, only the first term of the right side in Equation (9) can be used as follows:

CPR = 8.46 OD       (M)(9)

The CPR (M) is the carbonate precipitation rate in the BCS, while C (%) is the carbonate content in soil. The carbonate content (%) was defined in terms of the CPR by:

(10) $$C=\frac{m_{c}}{m_{s}}\times 100=CPR\frac{n}{\left(1-n\right)}\frac{\left(0.1\right)}{\rho_{s}}\times 100\;(\%)$$

where n is the porosity (in decimals), ρ_s_ is the density of soil particles (t/m^3^), and the units of the CPR are in M = mol/L. Equation (10) approximately gives C = 2.5%, when n = 0.4, CPR = 1.0 M, and ρ_s_ is 2.65 t/m^3^. Thus, it should be remembered that in an ordinary granular loose sandy soil, 1.0 M Ca^2+^ can produce approximately 2.5% of carbonates (calcite).

Substituting Equation (8) into Equation (10), the relationship between C and OD at 24 h precipitation can be obtained as:

(11) $$C=\frac{m_{c}}{m_{s}}\times 100=\{8.46OD-17.633{\left(OD\right)}^{2}\}\frac{n}{\left(1-n\right)}\frac{\left(0.1\right)}{\rho_{s}}\times 100\;(\%)$$

Assuming that the retardation effects with OD^2^ were negligible, because of the sufficient passage of time, the relationship between C and OD was given by:

(12) C=8.46W×OD(%)

and

(13) $$W=\frac{n}{\left(1-n\right)}\frac{\left(0.1\right)}{\rho_{s}}\times 100\;(\%)$$

**Figure 14:**
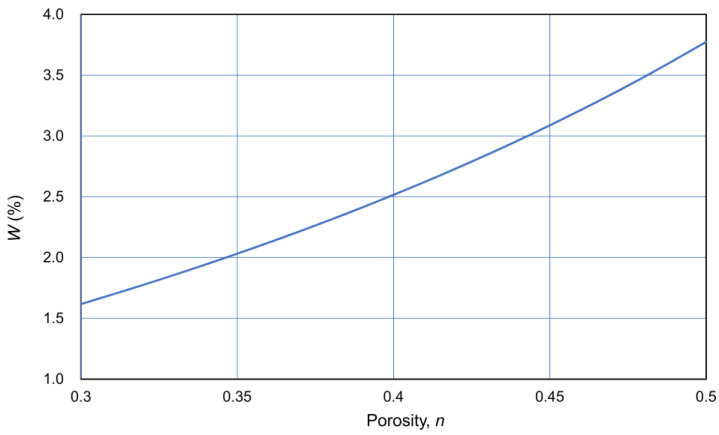
W−n relationship assuming ρ_s_ = 2.65 t/m^3^.

Assuming that the density of particles was 2.65 t/m^3^, W was expressed as a function of porosity n, as shown in Figure 14. Thus, C was expressed as a function of OD through the CPR.

From Equation (12), OD was expressed by:

OD = 0.118 C/W(14)

where OD is the viable OD which provides the standard OD−CPR relationship. Therefore, under the condition of bacteria-dependent MICP, which means no Ca^2+^ dependency, the distribution of bacteria represented by OD can be estimated from the C values measured with the gaseous pressure meter. Note that if OD* is needed, it is necessary to convert from OD to OD* by OD* = OD/Rcv. Thus, Equation (11) shows the relationship between OD (microscopic properties) and C (macroscopic properties), in which CPR, UCS, OD*, and Rcv values are all included. Therefore, the C value, which can be measured experimentally, can be one of the significant factors to anticipate the flow pattern of the BCS. The Ca^2+^ adsorbed by bacteria is adsorbed to the soil particles by Darcy’s law. It is noted that whether or not the flow pattern obeys Darcy’s flow is possibly related to the soil homogeneity.

## 5. Conclusions

One of the most important problems in the development of the MICP technique for engineering purposes is to introduce quantitative evaluation of urease-producing bacteria into chemical reactions. If this can be resolved, the test results can be comprehensively evaluated and consensus can be obtained. In this study, first, the method of quantitative evaluation of the bacteria was developed by establishing the standard OD−CPR relationship and the concept of experimental viability (Rcv). It was shown that the use of a bacterial evaluation method and Rcv can contribute to a comprehensive understanding of the properties and behavior of the MICP process. It was confirmed that the Ca^2+^/OD ratio controls the inhibition behavior in MICP. For example, if Ca^2+^/OD > 8.46 M in the BCS, then inhibition occurs, while at Ca^2+^/OD = 8.46 M, CPR = 8.46 OD and the CPR is proportional to the viable OD, Rcv, and OD*. As the CPR in MICP is determined to be time-dependent, when the elapsed time is not sufficient, then retardation in MICP will occur. Although the Rcv test takes two days, this is acceptable for engineering applications.

The UCS process due to carbonate precipitation can be divided into an initial rapid process (the first stage) due to the bonding of particle contacts and a gradual increase in the process (the second stage) after the first stage. In addition, it was shown that the first stage depends on the type of soil, especially the particle size, its distribution, the initial dry density, and the adsorbed intensity among bacteria, Ca^2+^ and the surfaces of soil particles with electrostatic charges. The a-value defined in this study is a good factor for quantitatively evaluating the UCS in “the first stage” in the MICP process.

Experimental work showed that the properties and behavior of MICP-treated soils are not solely dependent on carbonate content but are influenced by other factors. The factors affecting UCS, except for carbonate content, are mainly dry density governed by grain size, grain shape, and grain size distribution. Furthermore, it was indicated that the electrical surface charge density contributed to a very strong UCS.

The dry density and UCS relationship can be divided into two types of behavior, i.e., the first and second stages of the MICP process. The confusion between these two types of behavior will result in a misunderstanding of the MICP process.

## Figures and Tables

**Table 1:** Assumed constants of limestone and fine sand for comparison by a modified relative density.

	Specimen		Assumed		
	Shape	min. ρ_d_ (t/m^3^)	ρ_s_ (t/m^3^)	e_max_	e_min_
Limestone	Square Column	1.2	2.8	1.333	0
Biogrout and fine sand	Cylinder	1.4	2.65	0.893	0

**Table 2:** Soil properties used and a-values.

Type of Sand	Particle Density ρ_s_ (t/m^3^)	D_max_ (mm)	D_60_ (mm)	D_30_ (mm)	D_10_ (mm)	Uniformity U_c_	ρ_max_ (t/m^3^)	ρ_min_ (t/m^3^)	Initial C (%)	Initial ρ_d_ (t/m^3^)	a (kPa/%)
Vietnam sand	2.662	2	0.61	0.48	0.34	1.79	1.695	1.416	0.22	1.4	186–690
Coarse sand	2.65	2	1.35	1.05	0.87	1.55	1.568	1.336	0.2	1.28	34.5
Fine sand a	2.693	2	0.303	0.205	0.135	2.24	1.719	1.41	0.3	1.4	64.4
Medium sand	2.664	4.75	0.33	0.28	0.2	1.65	1.619	1.308	0.47	1.26	44
Fine sand b	2.679	4.75	0.295	0.195	0.13	2.27	1.683	1.377	0.2	1.38	53.9
Fine sand c	2.679	4.75	0.295	0.195	0.13	2.27	1.683	1.377	0.2	1.58	120
River sand	2.65	4.75	0.5	0.301	0.155	3.23	1.752	1.404	0.38	1.56	68.7

## Data Availability

The original contributions presented in this study are included in the article. Further inquiries can be directed to the corresponding author.
